# Recommendation by a law body to ban infant male circumcision has serious worldwide implications for pediatric practice and human rights

**DOI:** 10.1186/1471-2431-13-136

**Published:** 2013-09-08

**Authors:** Michael J Bates, John B Ziegler, Sean E Kennedy, Adrian Mindel, Alex D Wodak, Laurie S Zoloth, Aaron AR Tobian, Brian J Morris

**Affiliations:** 1 PO Box 29, Springwood 4127, Qld, Australia; 2 Department of Immunology & Infectious Diseases, Sydney Children’s Hospital, Randwick, Sydney, NSW 2031, Australia; 3 School of Women’s & Children’s Health, University of New South Wales, Sydney, NSW 2052, Australia; 4 Sydney Medical School, University of Sydney, Sydney, NSW 2006, Australia; 5 St Vincent’s Hospital and Kirby Institute, University of New South Wales, Sydney, NSW 2010, Australia; 6 Program in Bioethics and Medical Humanities, Northwestern University School of Medicine, Chicago, IL 60611-3015, USA; 7 Department of Pathology, School of Medicine, Johns Hopkins University, Baltimore, MD 21287, USA; 8 School of Medical Sciences and Bosch Institute, University of Sydney, Sydney, NSW 2006, Australia

**Keywords:** Circumcision, Infancy, Law, Ethics, Surgery, Public health, Religion, Tasmanian Law Reform Institute, American Academy of Pediatrics, Australia

## Abstract

**Background:**

Recent attempts in the USA and Europe to ban the circumcision of male children have been unsuccessful. Of current concern is a report by the Tasmanian Law Reform Institute (TLRI) recommending that non-therapeutic circumcision be prohibited, with parents and doctors risking criminal sanctions except where the parents have strong religious and ethnic ties to circumcision. The acceptance of this recommendation would create a precedent for legislation elsewhere in the world, thereby posing a threat to pediatric practice, parental responsibilities and freedoms, and public health.

**Discussion:**

The TLRI report ignores the scientific consensus within medical literature about circumcision. It contains legal and ethical arguments that are seriously flawed. Dispassionate ethical arguments and the United Nations Convention on the Rights of the Child are consistent with parents being permitted to authorize circumcision for their male child. Uncritical acceptance of the TLRI report’s recommendations would strengthen and legitimize efforts to ban childhood male circumcision not just in Australia, but in other countries as well. The medical profession should be concerned about any attempt to criminalize a well-accepted and evidence-based medical procedure. The recommendations are illogical, pose potential dangers and seem unworkable in practice. There is no explanation of how the State could impose criminal charges against doctors and parents, nor of how such a punitive apparatus could be structured, nor how strength of ethnic or religious ties could be determined. The proposal could easily be used inappropriately, and discriminates against parents not tied to the religions specified. With time, religious exemptions could subsequently be overturned. The law, governments and the medical profession should reject the TLRI recommendations, especially since the recent affirmative infant male circumcision policy statement by the American Academy of Pediatrics attests to the significant individual and public health benefits and low risk of infant male circumcision.

**Summary:**

Doctors should be allowed to perform medical procedures based on sound evidence of effectiveness and safety with guaranteed protection. Parents should be free to act in the best interests of the health of their infant son by having him circumcised should they choose.

## Background

Circumcision of male children in the English-speaking world became popular late in the 19th century because of a medical view at the time [1]. With the exception of the upper classes, it then declined in the United Kingdom after the 1940s when the National Health Service withdrew coverage for it, and in Australia began to decline in the 1970s because of a sudden change in pediatric policy that has continued to lack accordance with the ever-growing medical evidence [2]. In contrast infant male circumcision has remained popular amongst Americans of Anglo-Celtic heritage [1], which is also the predominant ethnic group in Australia. Currently, amongst Australian states and territories, infant male circumcision is least common in Tasmania and most common in Queensland, these being the coldest and most tropical states, respectively [3]. Nevertheless, the practice had until recently long been part of Tasmanian culture just as in the rest of Australia’s dominant Anglo-Celtic culture and Australia’s indigenous people.

The issue of circumcision of boys, especially in Anglophile countries, has come into sharp focus recently with the almost simultaneous release of a major new policy statement by the American Academy of Pediatrics (AAP) [4] and an extensive legal document by the Tasmanian Law Reform Institute (TLRI) [5]. The TLRI’s recommendations are not based on their own independent review of the evidence. In contrast, the AAP’s statement is a systematic compilation of the evidence and a thoughtful consideration of the relevant factors. While the TLRI report recommends that “*non*-*therapeutic*” circumcision of male minors be prohibited except where the parents have strong religious and ethnic ties to circumcision [5], the AAP report found (i) that the benefits of infant male circumcision exceed risks, (ii) that parents are entitled to factually correct, nonbiased information, (iii) that access to circumcision be provided for those families who choose it, and (iv) that third-party reimbursement is warranted [4]. The new AAP policy moves beyond its neutral policy in 1999 and it accords with another evidence-based policy assessment in Australia in 2012 that went further by calculating the risk-benefit (100:1 in favour) and finding that over their lifetime up to half of uncircumcised males were at risk of a medical condition caused by the foreskin [6]. These new pediatric policies are, however, at odds with statements by the British Medical Association [7], the Royal Dutch Medical Association [8], and the Royal Australasian College of Physicians (RACP) [9] advising against infant circumcision, and that, unlike the more recent ones, did not involve a scholarly literature-based review of the scientific evidence [2]. A claim in the RACP statement that it was evidence-based is untrue since, unlike the AAP policy statement, the authors of the RACP statement did not explain how they selected the literature used as the basis for their conclusions. Some poor quality observational studies, which did not support male circumcision, were cited while rigorous research, including randomized controlled trials and meta-analyses, supporting the practice were not cited. For these reasons, the RACP report should not be considered fair and balanced and should not guide policy [2]. New policies have been foreshadowed by the Centers for Disease Control and Prevention [10] and by the Canadian Paediatrics Association [11].

The TLRI report comes after an attempt in 2011 to ban the circumcision of minor male children in San Francisco, that was subsequently legislated against by a unanimous vote of the California Senate [12]; and a decision in 2012 by a minor court in Cologne which posed a potential threat to the legality of childhood male circumcision in Germany [13] leading the German Parliament to rule in favour of the practice [14]. The legislation upholding the legality of parents choosing to have their sons circumcised included a proviso that circumcision be performed by a trained professional in a safe environment. The wording suggested that any new law upholding circumcision in Germany would extend beyond religious reasons. The Jewish and Muslim communities vigorously and publicly opposed both attempted bans, arguing that anti-Semitic and anti-Islamic bias was responsible for these attempts. Thus the TLRI report may accord with the extremism associated with these highly publicized examples in the USA and Europe.

Because the TLRI report has the imprimatur of an academic law body it has attracted global attention. If adopted in Tasmania it could set a precedent for similar bans elsewhere. It therefore has significant potentially negative implications for pediatric practice and human rights worldwide. It may be no accident that the report originated in Tasmania. This small state of Australia has a predominantly Anglo-Celtic population, a very low rate of infant male circumcision [3], and few Jews and Muslims. This means that there would most likely be little opposition by the electorate to enactment of a ban on circumcision by the Tasmanian Parliament.

The present article argues that the views expressed in the TLRI report are extreme, impractical, at odds with evidence-based medical decision-making, a threat to good medical practice and public health, represent an attack on the medical profession and are of international importance.

## Discussion

### The TLRI report

The independent Commissioner for Children in Tasmania Mr Paul Mason, requested that the University of Tasmania MLaw student Warwick Marshall prepare an issues paper on non-therapeutic male circumcision. The issues paper was released in 2009 and called for submissions. Various medical and health experts, scientists and concerned parents made submissions pointing out the extensive medical benefits and low risk of this procedure, and the preference for infancy as the ideal time for male circumcision. These submissions, together with those from opponents, are referred to in the TLRI report [5]. Yet in formulating its recommendations the TLRI report in 2012 appears to have ignored the extensive scientific evidence supporting infant circumcision. It does, however, concede that *adult* male circumcision be allowed. The TLRI did not conduct an independent rigorous appraisal of the substantial medical literature on the topic of infant male circumcision, but confined itself to legal aspects. The TLRI premised their legal argument on a view of medical opinion that, “*No authoritative health policy maker in any jurisdiction with a frequency of relevant health conditions as low as that in Australia recommends circumcision as an individual or public health measure*.” The TRLI report opines in section 2.5.4, “*Without clarity in the application of the criminal law*, *those who perform*, *assist in or instigate a circumcision do so without knowing the extent to which they are protected from criminal liability*” [5].

The report nevertheless supports the circumcision of male minors for cultural and religious reasons. It is not, however, clear from the TLRI report how doctors are to decide whether parents are, or are not, sufficiently religious or sufficiently tied to an ethnicity which requires circumcision, nor which ethnicities should be considered an appropriate basis for such a parental decision. Nor is it clear how the operation of any laws developed from the TLRI recommendations would be monitored to ensure that they were not being used inappropriately. What would happen to a doctor whose judgement was considered incorrect by a court? The threat of criminal sanctions is very serious. Indeed, it would be a grave mistake for members of the medical profession to under-estimate the seriousness of the threat posed by the TLRI report. The uncertainty created places doctors in a predicament. Even more so when one considers that in Australia, as in the USA, only a minority of circumcisions are performed for religious reasons [15,16]. In this regard the TLRI report fails to acknowledge the rights of parents with atheist, agnostic or other religious beliefs to choose to have their baby boys circumcised for reasons such as health, hygiene, aesthetics or family tradition, especially given the increasing evidence that the benefits of infant male circumcision outweigh the risks, as indicated by the conclusions of the recent AAP policy statement [4]. It is perplexing that religious beliefs, but not medical evidence, should be allowed as the basis for decision-making by doctors and parents.

Other legal opinion differs from that which appears in the TLRI report. For example, a very respectable legal opinion, albeit not binding, was provided by a High Court Judge (and former Governor General of Australia), Sir William Patrick Dean, who stated that circumcision, “*for perceived hygienic* – *or even religious* – *reasons*” “*plainly lies within the authority of parents of an incapable child to authorize surgery on the basis of medical advice*” [17]. This statement dates to a time when the medical evidence in favour was not as strong as it is today. The legal case used by the TLRI has been misquoted in arguments to ban infant male circumcision, when in fact the case specifically dealt with *major* surgery (non-therapeutic *sterilization*) [18].

Article 24 (3) of the United Nations Convention on the Rights of the Child is not directed at abolishing infant male circumcision. Even some opponents of infant male circumcision do not support its criminalization, instead, in the case of religious circumcisions, insisting that it be performed in a medical setting by trained professionals [19-22].

At the time of writing the TLRI report had not been presented to the Tasmanian Parliament.

### The 2012 AAP policy

The AAP is regarded as an authoritative health policy maker internationally. The frequency of relevant health conditions in the USA and Australia are broadly similar. The AAP’s policy was developed by ethicists, epidemiologists and clinical experts, assisted by the Centers for Disease Control and Prevention, the American Academy of Family Physicians, and the American College of Obstetrics and Gynecology. The AAP policy graded the quality of the research the Task Force cited [4], as did the 2012 Australian report [6], and concluded that, “*Evaluation of current evidence indicates that the health benefits of newborn male circumcision outweigh the risks and that the procedure*’*s benefits justify access to this procedure for families who choose it*” [4]. It is not prescriptive. Instead, it states, “*Parents should weigh the health benefits and risks in light of their own religious*, *cultural*, *and personal preferences*, *as the medical benefits alone may not outweigh these other considerations for individual families*” [4]. Thus it retains the balance of rights and responsibilities between the individual child, the child’s parents, and society at large.

In contrast, a review article published at the same time as the AAP report appeared concluded, “*There is a lack of evidence both in favor of and against recommending routine neonatal circumcisions in the United States*” [23]. It questioned, “*whether we should continue unwarranted male circumcisions*, *especially when the major tenet of medical ethics is* ‘*do no harm*’.” The article failed to account for the substantial medical benefits of male circumcision reported during the previous five years [6,24-28], especially that from several high quality male circumcision trials [29]. Its reliance on the somewhat ambivalent 1999 AAP policy statement may explain its conclusion. Similarly, the TLRI’s comment that, “*no authoritative health policy maker in any jurisdiction with a frequency of relevant health conditions as low as that in Australia recommends circumcision as an individual or public health measure*” has now been made obsolete by the publication of the authoritative AAP policy in 2012.

### Ethics and human rights

Parents can legally authorize surgical procedures in the best interests of their children [4,30,31]. The AAP policy asserts that, “*As a general rule*, *minors in the United States are not considered competent to provide legally binding consent regarding their health care*, *and parents or guardians are empowered to make health care decisions on their behalf*[32]. *In most situations*, *parents are granted wide latitude in terms of the decisions they make on behalf of their children*, *and the law has respected those decisions except where they are clearly contrary to the best interests of the child or place the child*’*s health*, *well*-*being*, *or life at significant risk of serious harm*[33]”. Likewise consideration of internationally recognized rights of children results in a similar conclusion. The United Nations Convention on the Rights of the Child (UNCRC) 44/25 20 November 1989 held at Article 14 (2), “*States Parties shall respect the rights and duties of the parents and*, *when applicable legal guardians*, *to provide direction to the child in the exercise of his or her right in a matter consistent with the evolving capacities of the child*” [34]. Clearly for infants with no effective capacity, decisions are entirely the duty of the parents. Of course exceptions include failing to act in the interests of children or situations where a medical procedure or withholding a medical procedure causes serious harm. A recent critical analysis of the Australian government’s rationale for its vaccination policy argued that, “*vaccine choice* [*is*] *a human rights issue*” [35]. Vaccination is a minor medical procedure and is one that most parents choose for their children. Since the benefits of this intervention outweigh the risks and similarly failure to circumcise boys in a population will create a risk for future sexual partners, the vaccination of minors would appear to us to be analogous to the issue of the circumcision of boys. While a century ago US constitutional law has upheld the “*police power of the state*” in relation to compulsory vaccination [36], in democratic societies today neither vaccination nor childhood male circumcision are ever likely to be made compulsory.

Using as a basis of natural law and intuition, an articulate, albeit *prima facie*, argument has been made for a right to bodily integrity when it comes to circumcision [37]. Another author, by ignoring the substantive pediatric benefits [24,25], stated, “*the only significant* [benefits] (*reduced risk of penile cancer and sexually transmitted infections*) *do not apply until adulthood*” [38]. Claims that circumcision harms penile sensitivity [38] have no broad evidential support [39]. On the other hand, an ethicist has pointed out that, “*If circumcision is a net benefit to a child*, *parents do not violate his rights to bodily integrity or self*-*determination by circumcising him*” [40]. Another ethicist has provided compelling arguments in support of his contention that, “*appealing to this right* [to bodily integrity] *in the context of circumcision entails a misunderstanding of the nature of this right*” [41]. Since infant male circumcision is not prejudicial to the health of children, but instead is beneficial, it does not violate Article 24 (3) of the United Nations Convention on the Rights of the Child.

Further, the notion sometimes claimed that reducing parental choice advances human rights is contentious. Some argue that parental choice of circumcision for their infant son is illegitimate, because the choice can be made by the boy once he is an adult [38]. However, parents and physicians each have an ethical duty to the child to attempt to secure the child’s best interest and well-being [42]. Since the benefits outweigh the risks and the procedure is safe, there is no reason to single out circumcision for overriding parental choice. Indeed, an article from the UCLA School of Law stated that, “*a violations*-*only approach to human rights advocacy is unduly limiting*; *indeed it overlooks the duty of states affirmatively to create conditions necessary for the fulfilment of rights*. *In this case research now indicates that the availability of male circumcision* [for HIV prevention] *in some settings has the potential to serve as an important tool for realizing good health*” [43]. As stated by an ethicist, “*male infant circumcision falls within the prerogative of parental decision*-*making in the secular case and even more clearly in the religious case*” [41]. In a landmark review in 2004 Alanis and Lucidi point out that, “*Although the issue of informed consent promises to be at the forefront of any ethical*-*legal debate on circumcision*, *it is notable that a parent or legal guardian is bound to make countless other decisions for their growing child over the years until they are legally considered adults*, *many of which will likely have a more profound effect on them than the presence or absence of a foreskin*” [44].

Further undermining the argument for a unique right in relation to infant male circumcision is the fact that the timing of circumcision has a pronounced impact on both benefits and risks. Cultural and religious requirements of early circumcision aside, medical and practical considerations weigh heavily in favour of the neonatal period [25]. Surgical risk is minimized and the “*greatest accumulated health benefits*” are attained if circumcision is effected close to birth [4]. Benefits potentially lost include a significant reduction in urinary tract infections that in infancy may lead to kidney damage [24]. Delay may also result in increased cost, longer healing time, a requirement for temporary sexual abstinence, interference with education or employment, and loss of opportunity for, or delay in, the achievement of protection from sexually transmitted infections (STIs) for those who become sexually active early and for those who ignore advice on abstinence, thereby exposing them to increased risk of STIs, during the healing period [4,25]. At the same time, there are no long-term adverse effects of a successful medical circumcision on sexual function, sensitivity, sexual sensation or satisfaction [39]. It is, “*disingenuous to suggest that the procedure is comparable at both ages*” [45]. The latter ethical evaluation went on to point out, “*An adult cannot consent to his own infant circumcision*”. The author also referred to the fact that, “*Many nations that condemn circumcision are not as quick to condemn other comparably invasive and dangerous non*-*therapeutic procedures*” [45]. Examples of procedures performed on children that *are not medically* necessary include cosmetic orthodontia, correction of harelip, surgery for ankyloglossia, treatment of short stature by growth hormone injections and removal of supernumerary digits [45]. Given its substantive health benefits, it thus seems curious that circumcision seems unique among childhood procedures in attracting controversy [45].

The suggestion by the TLRI that childhood circumcision for religious or cultural reasons be permitted places such beliefs above the responsibility of parents to protect their son and his future sexual partners from the very real and high risk of adverse medical conditions from infancy through to old age [4,6].

The TLRI cites ethicists who believe that providing circumcision to minors violates their human rights [5]. But other ethicists, not cited by the TLRI, have argued that *denying* male circumcision violates ethical principles and human rights [46,47]. Using as a basis naturalism and intuition, an articulate, albeit *prima facie*, argument has been presented for a right to bodily integrity when it comes to circumcision [37]. The problems with this argument are (i) that it is pretextual, in that concerns with bodily integrity seem limited to male circumcision; (ii) bodily integrity *per se* is not generally considered as a fundamental right; and (iii) the international treaty frequently cited as the basis for this right of bodily integrity does not actually assert such a right [45]. Other authors, “*conjecture that misunderstandings about the* ‘*anatomical*’ *and the ordeal contribute to opposition to circumcision in Europe*” [48]. They argue that, “*from a cultural point of view*, *being circumcised opens opportunities and boosts autonomy more than it constrains them*.”

Religious and cultural reasons for infant male circumcision aside, Article 24 (1) of the UNCRC calls upon parties to the agreement to, “*recognize the right of the child to the enjoyment of the highest attainable standard of health and to facilities for the treatment of illness and rehabilitation of health*. *States Parties shall strive to ensure that no child is deprived of his or her right of access to such health care services*”. An ethicist refutes justice arguments that a fundamental right to bodily integrity exists warranting the abolition by the state of parental rights to have their son circumcised, pointing out that, “*neither the UNCRC nor the ethics literature provides an authoritative rule for resolving conflicts between rights*” [45]. The author goes on to say, “*Art*. *24*, §*3 does not in fact call for abolishing infant circumcision*. *First*, *its language does not do so*. *The net health effects of infant circumcision are positive*, *at least according to the AAP and WHO*. *If infant circumcision is not prejudicial to the health of children*, *it does not violate Art*. *24*, §*3*. *Second*, *Art*. *24*, §*3 never was intended to eliminate circumcision*. *Almost all Islamic states have signed or ratified UNCRC*, *as has Israel*. *They never would have agreed to the abolition of an essential practice of their established religions*. *In fact*, *one can construe Art*. *24*, §*3 to require circumcision*.” Article 24 (3) seeks to abolish, “*traditional practices prejudicial to the health of children*” [34]. Since infant MC is not prejudicial to the health of children, and in fact is beneficial, it does not violate Article 24 (3) [45]. On the other hand, since, “*abstention from circumcision is traditional in the UK and Scandinavia*”, the tradition in those countries, “*is conducive to transmission of various serious illnesses*, *including HIV*, *among sexually active minors*” [45]. As such, a tradition of non-circumcision could be considered as prejudicial to the health of children. The author then asserts that, “*Most parents care deeply for their children and try to do what is best for them*. *Parents generally are more concerned for their children than are activists who do not know the child but who find their parents*’ *choices distasteful*,” whereas parents who are opposed to infant male circumcision appear willing to, “*tolerate dissemination of an incurable disease to preserve the prepuce*” [45].

Ethicists who argue against childhood male circumcision typically base their arguments on a belief that male circumcision provides no medical benefit. We contend that the law has no place interfering in medical practice based on evidence, except to ensure that professionals always act responsibly. If the TLRI regards childhood male circumcision as inappropriate on medical grounds, why do they support it when carried out on religious grounds? The TLRI does not indicate how therapeutic male circumcision is to be differentiated from the non-therapeutic variety or who will determine the category.

The AAP policy implies that male circumcision should be routinely *offered* to parents of newborn sons in the expectation that some will accept while others will decline. Similar to the AAP policy, the 2010 policy of the RACP, despite its weaknesses [2], nevertheless states “*It is reasonable for parents to weigh the benefits and risks of circumcision and to make the decision whether or not to circumcise their sons*. *When parents request a circumcision for their child the medical attendant is obliged to provide accurate unbiased and up to date information on the risks and benefits of the procedure*. *Parental choice should be respected*. *When the operation is to be performed it should be undertaken in a safe*, *child*-*friendly environment by an appropriately trained competent practitioner*, *capable of dealing with the complications*, *and using appropriate analgesia*” [9]. The British Medical Association, in its guide on the law and ethics of male circumcision [7], recognizes the legality of male circumcision provided it is performed competently, is in the child’s best interests, and there is valid consent. It states that, “*circumcision of boys has been considered to be either medically or socially beneficial or*, *at least*, *neutral*,” but with the curious note that, “*the responsibility to demonstrate that non*-*therapeutic circumcision is in a particular child*’*s best interests falls to his parents*.” In the United Kingdom prevailing attitudes to vaccination and circumcision seem paradoxical [49]. The Royal Dutch Medical Association, while strongly opposed, nevertheless, “*fears that a legal prohibition would result in the intervention being performed by non*-*medically qualified individuals*”, which “*could lead to more serious complications*” [8].

### Criminality

In Common Law, the power of the State to interfere is derived from the doctrine of *Parens Patriae*, according to which the Crown has the ultimate responsibility for the welfare of incompetent persons. Whereas in American law the State interferes only when parental choice poses “imminent danger” to the child, in Common Law, the State is expected to take a position (even if not to impose it) whenever the welfare of a minor is at stake. This distinction looms large over the diverse ways in which controversial interventions, such as separation of conjoined twins and heart-transplantation, has been treated in American and British legal systems. It is evident that infant male circumcision, even if considered harmful by some, does not pose “imminent danger” to the baby.

By contrast the TRLI presents legal opinion not founded on accurate information. For example in 3.2.2 of the report, when invoking an uncertainty in respect of criminal law, the report asserts, “*The Code does not address when a parent*’*s authorisation can make the infliction of non*-*therapeutic harm to a minor lawful*” [5]. It is now known, however, that the provision of infant male circumcision results in benefits that outweigh the risks so it cannot be properly characterized as “harm” [4,6]. Without the faulty premise used in the report there are no grounds for its argument for uncertainty.

The TLRI report appears to take a less than cautious approach to interpreting legal authority when it holds at 3.3.2 that, “*it is likely that court authorization will be required when there is a heightened risk of a parent making a wrong decision as to whether a circumcision is in the child*’*s best interests*” [5]. The circumstances that may enliven mandated court authorization are stated as, “*parental disagreement*”, “*a greater than normal risk of complications occurring*” and “*the potential likelihood of the child making their own competent decision on the matter in the future*.” Whilst that statement may be literally correct the High Court of Australia, in explaining why sterilization does not come within, “*the ordinary scope of parental power to consent to medical treatment*”, held on page 237 that, “*As a starting point*, *sterilisation requires invasive*, *irreversible and major surgery*. *But so do*, *for example*, *an appendectomy and some cosmetic surgery*, *both of which*, *in our opinion*, *come within the ordinary scope of a parent to consent to*. *However*, *other factors exist which have the combined effect of marking out the decision to authorise sterilisation as a special case*. *Court authorisation is required*, *first*, *because of the significant risk of making the wrong decision*, *either as to a child*’*s present or future capacity to consent or about what are the best interests of a child who cannot consent*, *and secondly*, *because the consequences of a wrong decision are particularly grave*” [17]. Parental power to consent to medical treatment on behalf of a child diminishes gradually as the child’s capacities and maturity grow [17]. Accordingly, while it is impossible to know the exact boundaries where a procedure may be outside the normal scope of parental authority, when the above examples within parental authority are compared with circumcision (which is not major surgery), it is nevertheless clear that prophylactic circumcision falls safely within the ambit. In contrast, the TLRI report implies that childhood male circumcision for reasons other than religion may fall outside it.

In some ways, neonatal screening for genetic disorders presents comparable legal, ethical, public health and parental rights issues [50]. While medical practitioners are compelled to advise parents of the importance of screening, and most states have newborn screening statutes, these vary from being compulsory to *laissez faire*, allowing parents or guardians to refuse. An important difference is that, “*no newborn screening test involves a communicable disease*” [50]. Whereas there is no culture of refusal to screen neonates, there is a secular culture of opposition to infant male circumcision.

While the AAP’s policy provides guidelines, it did not seek the imprimatur of the law for its advice to its constituents, namely US pediatricians. Although its new guidelines are based on American experience, it should be appreciated that the practice, preventive health issues, culture and history of infant male circumcision in each jurisdiction are quite similar.

### Cost-effectiveness and access

While no doubt outside its ambit, the TLRI report is also of concern in terms of the economic implications. While only preliminary data are available in Australia, and then only for genital cancers [26], a recent, more extensive cost-effectiveness study of infant urinary tract infections and STIs found that if male circumcision rates in the USA were to decrease to the levels of 10% typically seen in Europe, the additional direct medical costs in infancy and later for treatment of these among 10 annual birth cohorts would amount to more than US$4.4 billion, even after accounting for the cost of the procedure ($291; range $146–437) and treatment of complications (average cost $185 each (range $130–235) and rate of complications of 0.4% (range 0.2–0.6%)) [51]. Notwithstanding the costs associated with the procedure and treatment of complications, each forgone infant circumcision procedure was estimated to lead to an average of US$407 in increased direct medical expenses per male and $43 per female [51].

In most US states Medicaid covers infant male circumcision for the poor. But 18 states have now withdrawn this provision in an environment of lobbying by opposition groups to do so. The decline has led to criticisms by public health advocates [29,52,53], since it is the poor who are being most adversely affected by conditions attributed to lack of circumcision. In a Medicaid birth cohort of 29,316, a recent study found that for HIV alone, “*for every year of decreased circumcision rates due to Medicaid defunding*, [the authors] *project*[ed] *over 100 additional HIV cases and* $*30*,*000*,*000 in net medical costs*” [54]. The study pointed out, “*The cost to circumcise males in this birth cohort at currently reported rates is* $*4*,*856*,*000*”. Considering the totality of medical conditions and infections that infant circumcision protects against [4,6], the cost savings would be greater than the savings for prevention of HIV infection. A modelling study of the consequences of Medicaid defunding found that, “*cost savings initially generated by non*-*coverage of elective circumcisions will be mitigated by the increasing rate and expense of medically indicated circumcisions*.” And that, “*These findings may have a significant impact on health policy*” [55]. The study only considered the increase in procedural costs for circumcision of boys aged 0–5 years. The lifetime costs for treatment of medical conditions associated with lack of male circumcision would therefore represent an even greater increase in the financial burden on healthcare systems, as discussed above.

In Australia, elective male circumcision is now no longer available in the public hospital system of any state or territory, i.e., is akin to the states in the USA that no longer provide Medicaid coverage for elective circumcision [52]. For circumcisions performed in private medical practices the charge generally levied vastly exceeds the Medicare rebate provided by the Commonwealth Government of Australia. The effect of the low rebate means that infant male circumcision is now unaffordable for low-income families. The AAP policy states, “*The preventive and public health benefits associated with newborn male circumcision warrant third*-*party reimbursement of the procedure*” [4]. This reinforces calls for a re-evaluation of parental access and funding for elective circumcision of their minor male children in all states in Australia [6,56] and, in the USA, the 18 states that no longer provide coverage under Medicaid [52,53]. There are also significant implications for policies in other countries as well. Together it further highlights why legislation acceding to the recommendations of the TLRI report would be regressive.

## Summary

We find the TLRI report to be unbalanced and not based on reasonable evidence. It poses a real or implied threat to the circumcision of male children not only in Tasmania, but other states and territories of Australia, as well as in other countries. The proposed legislative ban in Tasmania would, moreover, require a waste of public monies in remedying an imaginary problem and generating a result that would be unworkable. In no jurisdiction in the world are parental responsibilities to make choices in their children’s best interests usurped by legislation. This principle is supported by the United Nations Convention of the Rights of the Child. We submit that it would be imprudent to waste public money on an endeavor that: (i) is unnecessary, (ii) is unlikely to work based on the experience of other jurisdictions with less Draconian regulation, (iii) could be circumvented if it did work by a determined parent undertaking arduous travel with their baby boy to another state that at present permits circumcision in private practice. The trend in medical policy, economic considerations, and other matters point to the need for affirmative government policies for infant male circumcision. If parental choice is usurped when it comes to the desire of parents for circumcision of their male infants a flow-on could, moreover, extend to other interventions having medical benefits – the vaccination of children being a pertinent example. The Tasmanian Government should ensure certainty by swiftly rejecting the TLRI report. Not to do so poses a risk to public health, the rights of children to receive protection from adverse medical conditions over their entire lifetime, and human rights everywhere. A legislative ban in Tasmania would fuel the vigorous campaigning against childhood male circumcision by opponents in the USA, Europe, the UK and other countries. The rights of physicians (not legislators) to be the final arbiters of which medical procedures are to be offered and of parents to decide what is best for their child, should not be infringed. When legislators start dictating medical practice, the medical profession and society will be worse off.

## Competing interests

The authors declare that they have no competing interests.

## Authors’ contributions

MJB and BJM drafted the manuscript. BJM, MJB, JBZ, SEK, AM, ADW, LSZ and AART made substantial contributions to successive drafts and thereby the intellectual content of this article. All authors read and approved the final manuscript.

## Authors’ information

MJB is a solicitor in the Brisbane suburb of Springwood in Queensland, Australia. JBZ is Head of the Department of Immunology and Infectious Diseases and of the HIV Service at Sydney Children’s Hospital, Randwick, and an Associate Professor of Paediatrics at the University of New South Wales, in Sydney, Australia. SEK is a pediatric nephrologist at the Sydney Children’s Hospital, Randwick and Senior Lecturer in the School of Women’s & Children’s Health, University of New South Wales, Sydney, Australia. AM is Professor of Sexual Health Medicine, Sydney Medical School, University of Sydney, Sydney, Australia. ADW is an Honorary Consultant at St Vincent’s Hospital, Sydney, and a Visiting Fellow at the Kirby Institute for Infection and Immunity in Society, University of New South Wales, Sydney, Australia. LSZ is Professor of Medical Humanities & Bioethics and Religion, and Director of the Center for Bioethics, Science and Society, at Northwestern University School of Medicine, Chicago, Illinois, USA. AART is an Associate Professor in the Department of Pathology, School of Medicine, Johns Hopkins University, Baltimore, Maryland, USA. BJM is Professor Emeritus in the School of Medical Sciences and Bosch Institute at the University of Sydney, Sydney, Australia.

## Pre-publication history

The pre-publication history for this paper can be accessed here:

http://www.biomedcentral.com/1471-2431/13/136/prepub
